# Post-Adipose-Derived Stem Cells (ADSC) Stimulated by Collagen Type V (Col V) Mitigate the Progression of Osteoarthritic Rabbit Articular Cartilage

**DOI:** 10.3389/fcell.2021.606890

**Published:** 2021-03-22

**Authors:** Isabele Camargo Brindo da Cruz, Ana Paula Pereira Velosa, Solange Carrasco, Antonio dos Santos Filho, Jurandir Tomaz de Miranda, Eduardo Pompeu, Tiago Lazzaretti Fernandes, Daniela Franco Bueno, Camila Fanelli, Cláudia Goldenstein-Schainberg, Alexandre Todorovic Fabro, Ricardo Fuller, Pedro Leme Silva, Vera Luiza Capelozzi, Walcy Rosolia Teodoro

**Affiliations:** ^1^Rheumatology Division of the Hospital das Clinicas, Faculdade de Medicina, Universidade de São Paulo, FMUSP, São Paulo, Brazil; ^2^Bioterism Center of the Hospital das Clinicas, Faculdade de Medicina, Universidade de São Paulo, FMUSP, São Paulo, Brazil; ^3^Sport Medicine Division, Faculdade de Medicina, Institute of Orthopaedics and Traumatology of the Hospital das Clinicas, Universidade de São Paulo, FMUSP, São Paulo, Brazil; ^4^Hospital Sírio-Libanês, São Paulo, Brazil; ^5^Laboratory of Cellular, Genetic and Molecular Nephrology, Renal Division, University of São Paulo, São Paulo, Brazil; ^6^Department of Pathology of the Hospital das Clinicas, Faculdade de Medicina, Universidade de São Paulo, FMUSP, São Paulo, Brazil; ^7^Respiratory Medicine Laboratory, Department of Pathology and Legal Medicine, Ribeirão Preto Medical School, University of São Paulo (USP), São Paulo, Brazil; ^8^Laboratory of Pulmonary Investigation, Centro de Ciências da Saúde, Carlos Chagas Filho Biophysics Institute, Federal University of Rio de Janeiro, Rio de Janeiro, Brazil; ^9^National Institute of Science and Technology for Regenerative Medicine, Rio de Janeiro, Brazil

**Keywords:** adipose-derived stem cells, collagen V, osteoarthritis, experimental model, cartilage treatment, therapy, hyaline cartilage

## Abstract

Collagen is essential for cartilage adhesion and formation. In the present study, histology, immunofluorescence, morphometry, and qRT-PCR suggested that adipose-derived stem cells (ADSCs) stimulated by type V collagen (Col V) induce a significant increase of type II collagen (Col II) in the degenerative area of surgical-induced osteoarthritic rabbit articular cartilage (OA). *In vitro*, the effects of Col V on the proliferation and differentiation of ADSC were investigated. The expression of the cartilage-related genes *Col2a1* and *Acan* was significantly upregulated and *Pou5fl* was downregulated post-ADSC/Col V treatment. Post-ADSC/Col V treatment, *in vivo* analyses revealed that rabbits showed typical signs of osteoarthritic articular cartilage regeneration by hematoxylin and eosin (H&E) and Safranin O/Fast Green staining. Immunohistochemical staining demonstrated that the volume of Col II fibers and the expression of Col II protein were significantly increased, and apoptosis Fas ligand positive significantly decreased post-ADSC/Col V treatment. In conclusion, the expression of Col II was higher in rabbits with surgical-induced osteoarthritic articular cartilage; hence, ADSC/Col V may be a promising therapeutic target for OA treatment.

## Introduction

In humans, osteoarthritis (OA), which involves the degradation of joint cartilage and underlying bone, makes the collagen matrix disorganize and decreases the proteoglycan content within the cartilage (Sanchez-Adams et al., 2014). Without the protective effects of the proteoglycans, the collagen fibers of the cartilage can become susceptible to degradation and, thus, exacerbate the degeneration (MaroudasI, 1976). Inflammation of the synovium and the surrounding joint capsule can also occur. The articular changes cause pain, joint stiffness, and limited joint motion (Gu et al., 2017), strongly impacting the patient’s quality of life and the health system (Pasalini and Fuller, 2018).

Recent studies suggested that the irreparable injury of the cartilage is a major challenge in the treatment of human osteoarthritis and tissue engineering is considered to be an innovative and promising therapy for the patients (Wang et al., 2020; Kader et al., 2021; Tan et al., 2021; Xia et al., 2021). Among various cell therapies, adipose-derived stem cell (ADSC) therapy appears to hold promise (Bistolfi et al., 2021). ADSCs can differentiate into different mesenchymal cell types, including bone, cartilage, and adipose cells. Moreover, ADSCs are immune privileged due to their low immunogenicity. They can enhance the extracellular matrix microenvironment of the articulations by immune modulation of the inflammation and secreting cell growth factors, thus improving tissue repair and cartilage regeneration (Mazor et al., 2014; Wang et al., 2020). Nevertheless, the underlying mechanism is not fully understood.

Chondrocytes are specialized cells that compose the cartilage, synthesizing the collagenous extracellular matrix, an abundant ground substance that is rich in hyaluronic acid. They can be divided according to collagen types into (a) chondrocyte precursor cells (type I collagen), (b) differentiated hyaline chondrocytes (type II, IX, XI, and VI collagen), (c) hypertrophic chondrocytes (type X collagen), and (d) chondrocytes that modulate the synthesis of type I, III, and V collagen. Type II collagen (Col II) constrains the proteoglycans and the extracellular matrix responds to tensile and compressive forces that are experienced by the cartilage, with growth and remodeling of the extracellular matrix.

Type V collagen (Col V) promotes the adhesion and proliferation of chondrocytes and osteogenic cells, thus regulating cartilage adhesion. *Col Va1* mutations were detected in patients with osteoarthritis with mild chondrodysplasia (Yang et al., 2018). An interesting study suggested that targeting *Col Va2* promoted chondrocyte cell survival and extracellular matrix formation in steroid-induced necrosis of the femoral head (Yang et al., 2018). In addition, Col V is upregulated during adipogenesis and can stimulate adipocyte differentiation *in vitro* (Nakajima et al., 2002a,b). However, the stimulatory effect of Col V over ADSC proliferation and their ability to synthesize Col II in order to improve articular cartilage regeneration are unknown. Therefore, the present study evaluated the expression profiles of Col II in rabbits with surgical-induced osteoarthritic articular cartilage treated with adipose-derived stem cells (ADSCs) stimulated by Col V.

## Materials and Methods

### Animals and Ethics Statement

The animal care and experimental protocols used in this study complied with the ethical principles on animal experimentation, adopted by the Brazilian College of Animal Experimentation (COBEA) of animals used for scientific purpose, and were approved by the ethics committee in the use of animals from the Medical School of the University of São Paulo (Protocol No. 123/14). Twenty-four 15-week-old male New Zealand White rabbits with a mean weight ranging from 2.1 to 2.5 kg were used. The animals were individually housed in specific cages for rabbits, with environmental and food enrichment and under controlled conditions of temperature (room temperature of 22 ± 2°C) and artificial 12-h light–dark cycles. The animals were fed *ad libitum* with stander feed and water.

### Adipose-Derived Stem Cell Culture and Cell Differentiation

**FIGURE 1:**
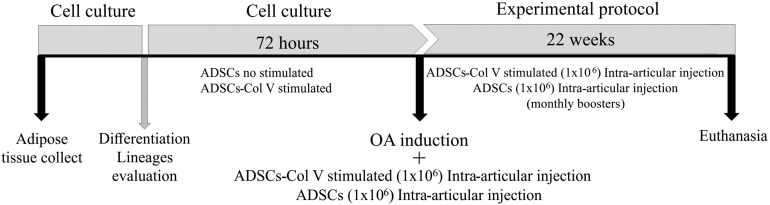
Schematic protocol of cell culture, OA induction, and treatment with Col V-stimulated ADSCs and ADSCs (Col V-unstimulated ADSCs). ADSCs, adipose stem cells; Col V, collagen V.

Sixteen rabbits were submitted to lipectomy by a longitudinal incision in the interscapular region to isolate the adipose tissue. To isolate ADSCs, adipose tissue from each animal was digested using Liberase (2.5 mg/ml) (Roche Diagnostics GmbH, Penzberg, Germany) for 40 min at 37°C and further centrifuged twice at 2,000 rpm for 5 min at 4°C. The precipitate was transferred to 25 cm^2^ culture flasks containing Dulbecco’s modified Eagle’s medium (DMEM; Gibco Life Technologies; Invitrogen, Paisley, United Kingdom) supplemented with 10% fetal bovine serum (Gibco^TM^) and 1% penicillin/streptomycin (Sigma Chemical Co., St. Louis, MO, United States). The culture was maintained at 37°C in an atmosphere of 5% CO_2_ until confluence. All experimental assays were realized in the third culture passage (Figure 1). The capacity to differentiate toward adipogenic, osteogenic, and chondrogenic lineages was tested using the differentiation kits StemPro^®^, (Gibco^®^, Life Technologies^TM^) according to the manufacturer’s recommendations. Cells were stained with Oil Red-O 0.5% (Sigma Chemical Co., St. Louis, MO, United States) in isopropanol 100%, Alcian Blue 1% in HCl 0.1 N, and Alizarin Red (Sigma Chemical Co., St. Louis, MO, United States) to evaluate adipogenic, chondrogenic, and osteogenic differentiation, respectively. Cell lineages were observed under a phase-contrast microscope (Nikon Eclipse TS100, Japan).

### Evaluation of Col II After Col V Stimulating ADSCs

Col V from human placenta (Sigma Chemical Co., St. Louis, MO, United States) was used to stimulate ADSCs. The ideal stimulus concentration of 50 μg/ml of Col V per 72 h was determined by a dose/response curve, using 25, 50, 100, and 200 μg/mL of Col V and control without collagen. The cell cultures were maintained for a period of 1, 2, 3, 7, 14, and 21 days. These cells were cultured over coverslips in DMEM (Gibco Life Technologies, Invitrogen, Paisley, United Kingdom) supplemented with 10% fetal bovine serum (Gibco^TM^) and 1% penicillin/streptomycin (Sigma Chemical Co., St. Louis, MO, United States) and were maintained at 37°C in an atmosphere of 5% CO_2_. The expression of Col II, a chondrogenic marker, was the criterion to determinate the ideal concentration and time of ADSC differentiation with Col V stimulus, evaluated by histomorphometry. To evaluate the potential of Col V-stimulated ADSCs, 2.5 × 10^5^ cells in the third culture passage were treated with Col V (50 μg/ml) per 72 h in 25 cm^2^ culture flasks with DMEM supplemented, in the same above conditions. After this period, cells were harvested with trypsin (Santa Cruz Biotechnology, Inc.), washed with DMEM, centrifuged at 2,000 rpm for 5 min in a conical tube (Falcon), and fixed successively in 4% paraformaldehyde and 70% alcohol. After 1 week, to evaluate Col II expression by immunofluorescence, the pellet was embedded in paraffin and 5 μm sections adhered to the slides, previously treated with 3-dimethylamino silane, dewaxed in xylene, and rehydrated with alcohol in decreasing concentrations, running and distilled water, and PBS. After blocking with 5% BSA, the sections were digested with pepsin (Sigma Chemical Co., St. Louis, MO, United States) 4 mg/ml in 0.5 N acetic acid for 30 min at 37°C. They were then washed in PBS and incubated with mouse polyclonal antibody anti-Col II (1:50, Abcam Inc., Burlingame, CA) overnight at 4°C. Afterward, the specimens were washed in PBS–Tween_20_ and incubated with goat anti-mouse IgG antibody-Alexa Fluor 488 (1:200) with DAPI (1:200), a nuclear dye. The reaction was visualized utilizing confocal microscopy (ZEISS LSM 510 Meta/UV) and quantified by image analysis software (see below).

### *Col2a1, Acan*, and *Pou5f1* Gene Expression

The ADSCs were seeded in 75 cm^2^ culture flasks and divided into three groups: stimulated with Col V (50 μg/ml) for 72 h; TGF-β1 (10 ng/ml) for 72 h, a growth factor widely used to induce cellular differentiation; and without stimulus. Total RNA was extracted by the method of Chomczynski and Sacchi (1987) using the TRIzol reagent and RNA samples were treated with the kit RQ1 RNase-Free DNase (Promega^®^, Thermo Fisher Scientific, Rockford, IL), according to the manufacturer’s instructions. RT-PCR reactions were performed with the primers drawn to *Col2a1* (α1 chain Col II), *Acan* (aggrecan), and *Pou5f1* (POU domain, class 5, transcription factor 1, protein transcription factor involved in the process of self-renewal and maintenance of stem cells) (Table 1).

**TABLE 1:** Oligonucleotide sequence.

Gene	Sense	Reverse	Base pair (bp)
*Gapdh*	AGGTCATCCA CGACCACTTC	GTGAGTTTCC CGTTCAGCTC	202
*Pou5f1*	GCCGACAACA ATGAGAACCT	ACACGGACCA CGTCTTTCTC	197
*Acan*	GTGACCGAGGT CAGTGGATT	CCAGGTCAGGG ATTCTGTGT	175
*Col2a1*	GAGACCTGAAC TGGGCAGAC	GACACGGAGT AGCACCATCG	192

**Col2a1, α1 chain Col II; Acan, aggrecan; Pou5f1, POU domain, class 5, transcription factor 1; Gapdh, glyceraldehyde 3-phosphate dehydrogenase, internal control.**

Gene expression was evaluated using the Real-Time PCR System (Applied Biosystems, Foster City, CA, United States) with the kit SuperScript III Platinum SYBR^®^, Green One-Step qRT-PCR (Life Technologies). The cycling conditions for the genes were as follows: 50°C for 10 min (for cDNA synthesis) followed by 35 cycles of 95°C for 15 s, 60°C for *Gapdh* and *Col2a1*, 59°C for *Acan*, and 57°C for the *Pou5f1* gene for 30 s and 72°C for 30 s. The analysis was performed by the 2^–Δ^ ^Δ^ ^*CT*^ method using *Gapdh* gene as an internal control.

### Col V-Stimulated ADSC Culture to Therapeutic Assays

After Col V stimuli evaluation, ADSCs in the third culture passage were cultivated with Col V (50 μg/ml) per 72 h in 75 cm^2^ culture flasks with DMEM (Gibco Life Technologies, Invitrogen, Paisley, United Kingdom), supplemented with 10% fetal bovine serum (Gibco^TM^) and 1% penicillin/streptomycin (Sigma Chemical Co., St. Louis, MO, United States), at 37°C in an atmosphere of 5% CO_2_. The Col V-stimulated ADSCs were cryopreserved in DMEM supplemented with 10% fetal bovine serum and 10% dimethyl sulfoxide (DMSO; Sigma Chemical Co., St. Louis, MO, United States) at −80°C. To therapeutic protocol, the ADSCs viability was evaluated with Trypan blue staining in Neubauer camera and 1 × 10^6^ cells were diluted in 0.3 mL of the Physiological solution.

### Induction of Osteoarthritis in Rabbits

Animals (*n* = 24) were anesthetized intramuscularly with xylazine (5 mg/kg) and ketamine (50 mg/kg). The procedures were performed under aseptic conditions. The induction of experimental OA was performed through a lateral parapatellar incision in the right knee followed by the removal of an anterior fourth of the lateral meniscus, preserving the ligaments, according to Moskowitz et al. (1973), with modifications. For the suture, a 4.0 mononylon wire was used at single-spaced points. All animals were given antibiotic (enrofloxacin 5 mg/kg, once daily) for 3 days and an anti-inflammatory (ketoprofen 10 mg/kg, 12/12 h), subcutaneously.

### Therapeutic Protocol and Experimental Groups

Following the procedure of OA induction and antisepsis of the right knee with iodized alcohol, the OA/ADSC (*n* = 8) and OA/ADSC/Col V (*n* = 8) groups were, respectively, treated with autologous intra-articular injections of 0.3 ml of 1 × 10^6^ ADSCs and 0.3 ml of 1 × 10^6^ ADSCs/Col V, which were previously cultured with Col V (50 μg/ml for 72 h). After the cell administration, stretching and flexion movements were made in the knee joint in order to disperse the suspension in the intra-articular space. This treatment was performed monthly for a period of 22 weeks (Figure 1). The OA (*n* = 8) group received no treatment.

### Histological Analysis

The right (operated) and the left (contralateral unoperated) knee joints of all animals were collected and fixed in 10% buffered formalin about 24 h followed by decalcification with 7% aqueous nitric acid. Then, histological procedures were made for morphological analysis and histological sections (4–5 μm thickness) were stained with H&E and Safranin O/Fast Green.

#### Semiquantitative Evaluation of Joint Injury

The following graduation, modified from the OARSI joint injury grade (Pritzker et al., 2006; Pearson et al., 2011; Waldstein et al., 2016), was applied to score the joint injury: *grade 0* = when the cartilage surface is smooth; the matrix and chondrocytes are organized into superficial, mid, and deep zones; and the cartilage morphology is intact; *grade 1* = when the surface is intact, but with irregularity of superficial fibrillation cluster proliferation and the mid and deep zones are unaffected; *grade 2* = when the surface is discontinuous with deep fibrillation, accompanied by cell proliferation and disparity in matrix staining or cell death; *grade 3* = when vertical fissures extended vertically until the mid-zone accompanied by cell death and cluster proliferation; *grade 4* = when the cartilage is corroded with loss of the cartilaginous matrix, cyst formation within the cartilage matrix, and cluster proliferation; *grade 5* = when the matrix is exposed and hyaline cartilage is unmineralized and completely eroded; the bone plate with microfracture and reparative fibrocartilage occupying gaps in the surface; and *grade 6* = when the matrix is deformed, with microfracture, fibrocartilaginous, and osseous repair extending above the previous surface. The resulting changes in the contour of the articular surface are illustrated in Supplementary Figure 1.

### Immunohistochemical Analysis

The characterization of chondrocyte apoptosis and Col II expression was assessed, respectively, by immunoperoxidase (IP) and immunofluorescence (IF). Tissue sections (4 mm thick) were cut from formalin-fixed, paraffin-embedded blocks containing representative cells and tissue and processed for immunohistochemistry (IHC).

#### Immunoperoxidase

The chondrocyte apoptosis in the articular cartilage was evaluated by immunohistochemistry for Fas ligand using the mouse anti-FasL monoclonal antibody (1:20, Neomarkers, Fremont, CA) and the Novolink development kit (Leica Biosystems, New Castle Ltd., United Kingdom), according to the manufacturer’s specifications.

#### Immunofluorescence

The cartilage sections on slides were deparaffinized in xylene-alcohol and rehydrated in running water, distilled water, and phosphate buffered saline. Firstly, enzymatic digestion with chondroitinase ABC 2UN (Sigma Chemical Co., St. Louis, MO, United States) in buffer containing 50 mM Tris, pH = 8.0, sodium acetate 60 mM, and 0.02% BSA at 37°C for 3 h was performed for exposure and recovery of antigenic sites. Subsequently, antigenic recovery was done with 8 mg/ml porcine pepsin (Sigma Chemical Co., St. Louis, MO, United States, 10,000 UTI/ml) diluted in acetic acid 5 N for 30 min at 37°C. For blocking non-specific sites, the sections were incubated with 5% BSA in PBS for 30 min. Polyclonal mouse anti-Col II antibody (1:20; Abcam, Burlingame, CA, United States) was incubated overnight at 4°C. After a PBS 0.05%–Tween_20_ washing cycle, goat anti-mouse IgG antibody (Alexa Fluor 488, Invitrogen, Life Technologies) diluted (1:150) in PBS and 0.006% Evans blue was incubated at room temperature for 60 min. After the sections were washed and mounted in glycerin-PBS (v/v), they were analyzed using an Olympus BX51 fluorescence microscope (Olympus Corp., Tokyo, Japan).

### Morphometric Analysis

The quantification of histologic, immunoperoxidase, and immunofluorescence parameters was done by digital imaging through the Image-Pro Plus 6.0 software. Briefly, the image analysis system consisted of an Olympus camera (Olympus Corporation, St Laurent, Quebec, Canada) coupled to an Olympus microscope (Olympus BX51), from which the images were sent to an LG monitor by means of a digitizing system (Oculus TCX, Coreco Inc., St Laurent, Quebec, Canada) and downloaded to a computer (Pentium 1330 MHz). Ten images from the culture were processed with the software (Image-Pro Plus 6.0).

The histologic sections of femoral condyles and tibial plates stained with H&E were scanned at ×200 magnification to evaluate cartilage thickness. Five vertical lines were drawn from the cartilage surface to the tidemark, starting from a central point of the tissue and 250 and 500 mm to the right and to the left. The cartilage thickness was calculated in μm^2^ from the arithmetic mean of the drawn lines. The density of the chondrocyte per cartilage area was evaluated by the stereological point-counting method developed by Gundersen et al. (1988) with modifications. Using the measurement tools of the Image-Pro Plus 6.0 software, a reticulum with 100 points orthogonally distributed over the acquired image was constructed. The cells coincident with the points in the reticulum were counted and the result was given as percentage of chondrocyte cells per mm^2^.

The proteoglycan levels in the cartilage were evaluated based on the quantification of the Safranin O/Fast Green staining intensity using the Image-Pro Plus 6.0 software (Pastoureau et al., 2010). Ten images in ×400 magnification were acquired and the staining intensity was measured by the software. The mean of the area stained in blue was divided by the mean of the total area analyzed, and the result expressed as percentage per mm^2^.

The Col II immunofluorescence in cultures and tissue was analyzed through images acquired at ×400. The area of each field analyzed was measured in μm^2^ and the mean of the immunostaining area corresponding to Col II was divided by the mean of the total area analyzed and the final result was expressed as percentage of fibers/mm^3^.

### Statistical Analysis

Statistical analysis was performed using GraphPad Prism 4.0 software (GraphPad Prism, Inc., San Diego, CA, United States). One-way analysis of variance (ANOVA) with Newman–Keuls post-test for comparison between groups or Kruskal–Wallis test followed by multiple Dunn’s test for non-parametric data was used. Results are expressed as mean ± standard errors of the mean (SEM); a *P* < 0.05 was considered statistically significant.

## Results

### ADSC Differentiation

**FIGURE 2:**
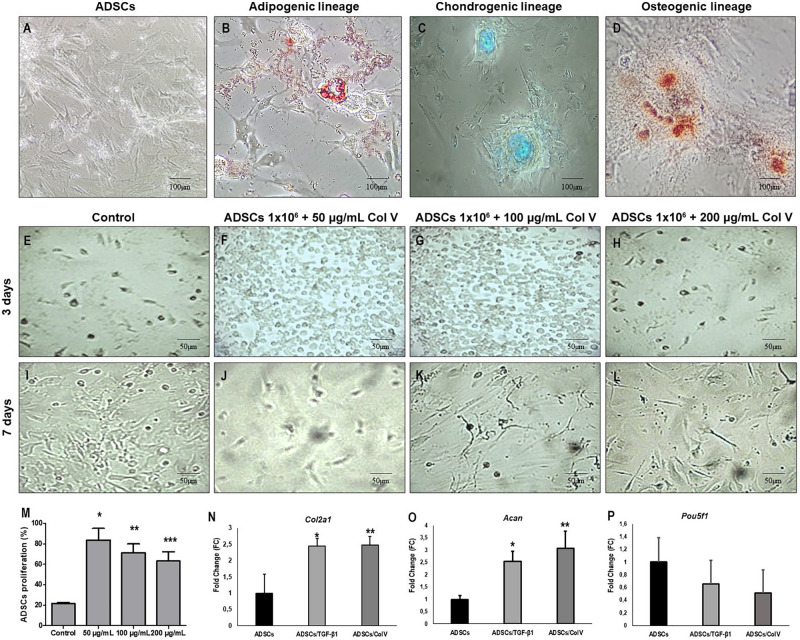
ADSCs grown in specific medio and differentiation into adipogenic, chondrogenic and osteogenic lineages. Adipogenic lineage presents cytoplasmic lipid droplets red stained with Oil Red **(B)**, chondrogenic lineage shows matrix proteoglycans production identified by Alcian Blue staining (blue) **(C)** and osteogenic lineage exhibit calcium salts identified by Alizarin Red **(D)**. Control ADSCs grown in DMEM **(A)**. The proliferation of ADSCs **(E–M)** and expression of chondrogenic related genes and were increased post-ADSCs/Col V and TGF-β treatment. The expression of *Col2a1* **(N)**, *Acan* **(O)** and Poup5f1 **(P)** at the mRNA level was measured by RT-PCR after 3 and 7 days of incubation in the chondrogenic medium of ColV and TGF-β treated ADSCs. The cell viability of ADSCs was detected by CCK-8. ADSCs, adipose stem cells. The experiments were performed in triplicate. *P < 0.05; **P < 0.001; ***P < 0.0001.

ADSCs grown in specific differentiation media were able to differentiate into adipogenic, chondrogenic, and osteogenic lineages, characterized, respectively, by the presence of cytoplasmic lipid droplets (Oil Red), synthesis of proteoglycans by the presence of a proteoglycan-rich matrix (Alcian Blue), and aggregates of calcium salts with calcium deposits (Alizarin Red) (Figures 2A–D).

### Col V Enhances ADSC Proliferation and Increases the Expression of Chondrogenic Genes *in vitro*

The impact of Col V in ADSC proliferation was assessed (Figures 2E–L). After incubation with Col V 50 μg/ml for 3 days, the proliferation assay revealed that Col V enhanced the proliferation of ADSCs in a dose-dependent manner (Figure 2M). Moreover, the expression of chondrogenic-related genes was measured by real-time qPCR. As expected, the expression of *Col2a1* and *Acan* genes was significantly upregulated post-Col V/ADSC incubation after chondrogenic differentiation at days 3 and 7 (both *P* < 0.05, Figures 2N,O), whereas *Pou5f1* gene expression showed a tendency to be downregulated (*P* = 0.07, Figure 2P). A similar expression of *Col2a1* and *Acan* genes was found with TGF-β1, a growth factor that stimulates ADSCs (Figures 2N–P).

### The Expression of Col II Was Increased During the Process of Col V/ADSC Treatment *in vitro* and *in vivo*

**FIGURE 3:**
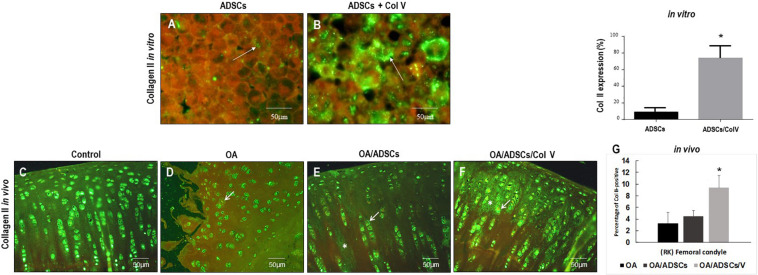
Representative images of the strong green birefringence by immunofluorescence of Col II after 3 days with Col V (50 mg/ml) during the process of Col V/ADSC treatment *in vitro* **(A,B)** and *in vivo* **(C–F)**. The **(G)** shows the representative graphics of the amount of Col II expression *in vitro* and *in vivo* evaluated by Image-Pro Plus 6.1 software. *P < 0.05.

Figures 3A–G show the immunofluorescence of Col II *in vitro* and *in vivo* post-ADSC treatment stimulated with Col V for 3 days (50 μg/ml). A strong and diffuse green birefringence of Col II was observed in the pellets of ADSCs/Col V (Figure 3B), contrasting with the weak and focal birefringence in ADSCs (Figure 3A). These features coincide with the significant increase of Col II expression in ADSCs/Col V when compared with ADSCs (*P* < 0.01) (Figure 3C). A similar strong and diffuse green birefringence of Col II was observed in osteoarthritic rabbit articular cartilage post-ADSCs stimulated with Col V intra-articular injection (Figure 3F). Of note is the progressive increase of Col II expression from osteoarthritis (Figure 3D) and OA/ADSCs (Figure 3E) to osteoarthritis being maximum and significant in OA/ADSCs/Col V (*P* < 0.05).

### Post-ADSC/Col V Treatment Avoids the Progression of Osteoarthritic Rabbit Articular Cartilage

**FIGURE 4:**
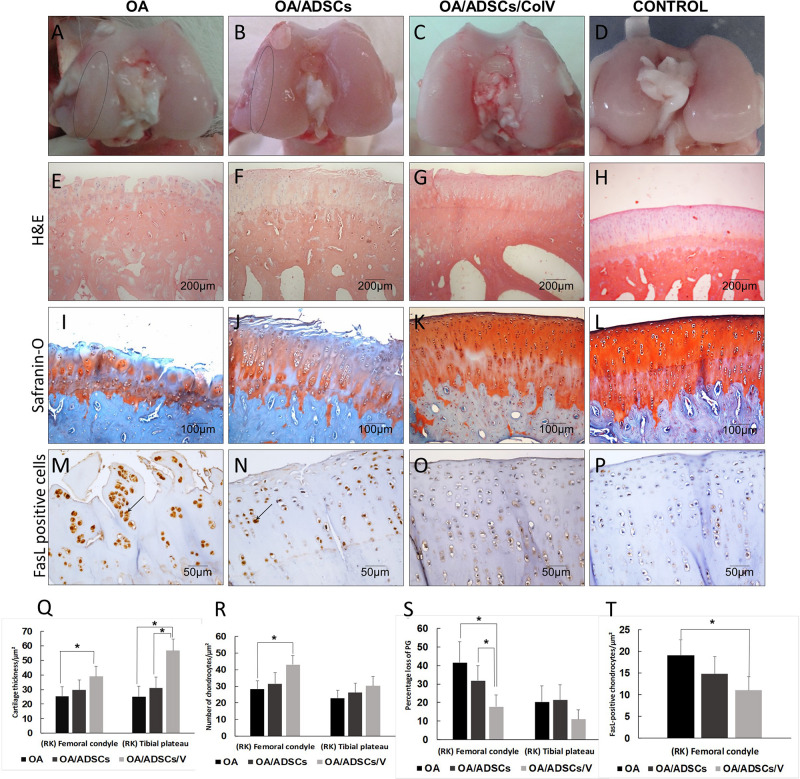
The morphogenic changes showed typical signs of osteoarthritic rabbit articular cartilage regeneration post-ADSCs/Col V. **(A–D)** Coronal section of representative femoral heads. The circle indicates the femoral condyle cartilage degeneration. Hematoxylin and eosin **(E–H)** and Safranin O/Fast Green **(I–L)** staining. Chondrocytes apoptosis Fas ligand+(arrows) **(M–P)**. Morphometric analysis showing cartilage parameters of the upper outer subchondral bone of the femoral heads **(Q)**, chondrocytes **(R)**, proteoglycans **(S)**, and apoptosis **(T)**. *P < 0.05.

Figure 4A shows the gross characteristics of the femoral condyle from the OA group including thinning of the cartilage and having a pearly white color and several stretch marks of chondral erosions in the cartilage. The lesions were less severe in the OA/ADSC group presenting cartilage with superficial erosions and focal stretch marks of chondral erosions (Figure 4B). These gross characteristics contrast with the bright white color and regular surface of the femoral chondral cartilage in the joints of OA/ADSC/Col V animals (Figure 4C), similar to control (Figure 4D).

**FIGURE 5:**
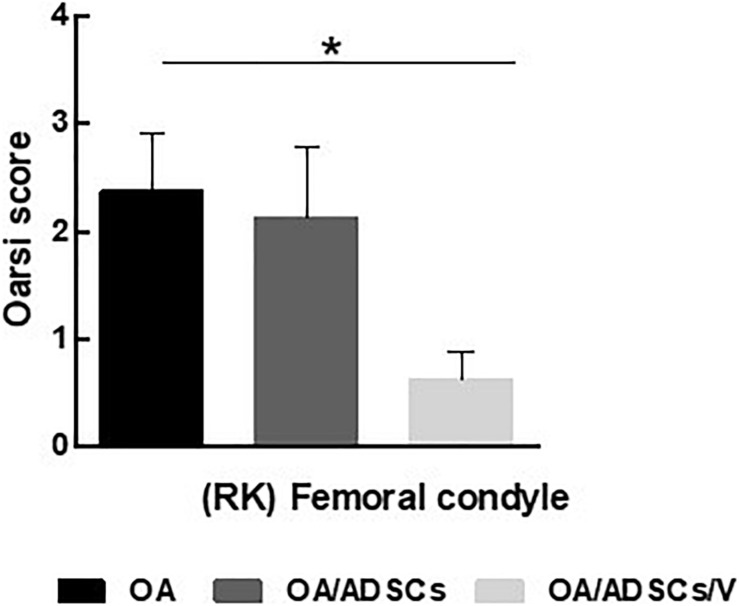
OARSI score grading for histologic alteration in OA, OA/ADSCs, and OA/ADSCs/Col V. *P < 0.05.

Histologically, the OA group coincided with severe stretch marks and thinning cartilage, osteophyte formation, subchondral sclerosis, cellular disorganization of chondrocytes and reduction of bone trabeculae, beyond complete tidemark discontinuity (Figure 4E), and weak Safranin O/Fast Green staining intensity of the extracellular matrix indicating reduction of glycosaminoglycans (Figure 4I). The OA/ADSC joints show less severe osteoarthritic histologic changes compared with those of the OA group with superficial fibrillations, moderate cellular disorganization, maintenance of the tidemark, and subchondral bone with less sclerotic aspect (Figure 4F). In this group, Safranin O/Fast Green staining intensity was moderate, indicating minor loss of proteoglycan content (Figure 4J). In contrast, the OA/ADSC/Col V group showed only stretch marks of the cartilaginous surface with recovery of cartilage thickness (Figure 4G), minor cellular disorganization, continuous tidemark, subchondral bone without sclerotic aspect (Figure 4G), and maintenance of proteoglycan content compared with the OA and OA/ADSCs (Figures 4I–K), similar to control cartilage (Figures 4H,L). Supplementary Figure 1 shows the injury grades in OA, OA/ADSCs, and OA/ADSCs/Col V. The OA/ADSCs/Col V presented a significant cartilage regeneration in relation to OA (Figure 5; *P* = 0.019).

These histological changes in the rabbits’ joints from the OA/ADSC/Col V group, which had been injected with ADSCs/Col V, coincide with the significant increase of cartilage thickness (*P* < 0.05; Figure 4Q), chondrocyte density (*P* < 0.05; Figure 4R), proteoglycans repair (*P* < 0.05; Figure 4S), and apoptosis reduction (*P* < 0.05; Figures 4M–P,T). Regarding the OA/ADSC group, intra-articular injection of ADSCs was also able to significantly increase chondrocyte density and cartilage thickness, and there was reduction of apoptosis than the amount observed in rabbits from the OA group but much less than that from the ADSC/Col V group.

## Discussion

Under the conditions of the present study, we found plenty of associated effects in rabbit OA. They included the following: (1) Col V enhances rabbit ADSC proliferation and differentiation and increases *in vitro* Col II expression in ADSCs; (2) in the *in vitro* expression of cartilage-related genes, *Col2a1* and *Acan* were significantly upregulated and *Pou5fl* was downregulated post-ADSC/Col V treatment; and (3) *in vivo*, the injury grades post-ADSC/Col V treatment showed typical signs of osteoarthritic articular cartilage regeneration, including decreased cartilage thickness, increased number of chondrocytes, decreased proteoglycan loss, decreased number of apoptotic chondrocytes, and increased expression of Col II protein. The current study showed that ADSCs stimulated by Col V (ADSCs/Col V) induced a significant regeneration of cartilage, indicating that ADSCs/Col V may be a therapeutic target for the treatment of osteoarthritis.

The experiments in the present study were performed in a surgical-induced osteoarthritic rabbit articular cartilage, established by Moskowitz et al. (1973) and modified by Velosa et al. (2007), which reproduces progressively the morphological changes found in human OA. The established period of 22 weeks was described equivalent to an advanced time of disease, with macroscopic lesions of the cartilage and histology features, characterized by cell disorganization, presence of cell clones, fibrillations, and fissures, in addition to loss of collagen and proteoglycans (Velosa et al., 2007).

Regarding the concentration of Col V used to stimulate ADSCs, the treatment time, the number of cells administered, and the route of administration, we employed a therapeutic protocol based on a monthly intra-articular administration of ADSCs 10^6^/Col V 50 μg/ml, which is the ideal stimulus concentration based on cell culture proliferation and expression of Col II, a chondrogenic marker, during a 22-week period (Velosa et al., 2007). Intra-articular administration was chosen considering that the cells will be applied in a joint in a closed environment. Moreover, it simulates clinical practice. In addition, we decided not to perform the treatment with a shorter interval, considering that some studies detected mesenchymal stem cells (MSCs) up to 1 month after intra-articular injection (Satué et al., 2019).

The balance between the synthesis and degradation of matrix proteins in the extracellular matrix (ECM) is crucial for the dynamic stability of tissues (Humphrey et al., 2014). The interaction of chondrocytes with the cartilage ECM plays an important role for chondrocyte anchorage, proliferation, differentiation, and function (He et al., 2014). In cell culture, chondrocytes have the ability to synthesize a large variety of matrix proteins, including collagen types I, II, III, IV, and V and fibronectin (Aigner et al., 1993). Col V is synthesized and deposited in the ECM, where it cross-linked with Col I and III, to form the collagenous matrix skeleton (Mak et al., 2016). By histological and proteomic analyses, Hong et al. (2010) reported that Col V in the ECM improved cell distribution and actin fiber frame promoting adhesion in the initial stage of chondroblast differentiation. The growth and remodeling of the cartilage matrix may revert morphologic injury. Moreover, the present study showed that the gene and protein profile of Col II was significantly higher in OA rabbits, and its upregulation was confirmed both *in vitro* and *in vivo*. These findings suggest that the balance between Col V and Col II expression may revert to the pathogenesis of OA.

Chondrocytes are the only cell type composing the articular cartilage and encompass only 2–5% of its compartment, the remaining being occupied by a hydrous ECM of collagens (mostly Col II) and proteoglycans. The main function of chondrocytes is to preserve the integrity of the cartilage by reverting injury to the matrix. Nevertheless, in OA, the biomechanical and biochemical microenvironment of the cartilage is altered, as well as in the performance of chondrocytes. These collateral changes are considered in part due to repression of chondroblast differentiation from MSCs and apoptosis of chondrocytes (Hwang and Kim, 2015). At early stages, OA is mainly characterized by the apoptosis of chondrocytes, then the reparative reaction of the cartilage is initiated. The imbalance between metalloprotease-mediated cartilage resorption and chondroblast-mediated cartilage restoration led to distorted histoarchitecture and progressive breakdown of joints. As the cartilage is the main affected component in OA, molecular studies of OA have focused on the mechanism of damage to the articular cartilage (Bertrand et al., 2010). Cartilage degeneration in OA is a progressive process complemented with the gradual loss of Col II and a gradual decrease in mRNA expression of *Acan* and *Col2a1* (Buckwalter et al., 2004; Brew et al., 2010). As expected, we found that the gene expression of the chondrocyte markers *Acan* and *Col2a1* was increased after ADSC/Col V treatment. In contrast, we found decreased expression of *Pou5f1 gene* post-ADSC/Col V treatment. Previous studies have reported that *Pou5f1* works in maintaining the cancer stem cell fate of osteosarcoma (Siclari and Qin, 2010). Guo et al. (2017) demonstrated that *Pou5f1* gene expression was downregulated by miR-335, and suggested that it could repress *Pou5f1* expression by post-translational regulation. They also found that cells expressing miR-335 possessed decreased stem cell-like properties. Although *Pou5f1* has not been reported in OA pathology, we infer that *Pou2f1* might also be a candidate gene connected with OA pathology and ADSC/Col V treatment.

The mechanism of action of Col V in the stimulation of ADSCs to synthesize Col II is unknown. However, based on our results, we infer that the biochemistry characteristics and biologic functions of Col V may induce cell proliferation and differentiation of ADSCs to produce collagen as previously reported (Ruggiero et al., 1994; Mak et al., 2016). Collagen type V works as crucial components for preserving the structural integrity of the cartilage skeleton, promoting adhesion and proliferation of several cell types including fibroblasts and chondrocytes (Fichard et al., 1995; Breuls et al., 2009; Wu et al., 2009). These properties of cell adhesion, migration, and proliferation are primarily due to tripeptide sequence consisting of arginine, glycine, and aspartic acid (RGD) sequences present in greater quantity in the α2(V) chain that bind primarily to the membrane integrins and could stimulate ADSC proliferation and differentiation (Ruggiero et al., 1994, 1996). Col V adhesion may also occur through a 30-kDa α1(V) chain fragment containing a pool of basic amino acid residues with affinity to heparin and heparin sulfate, which could enhance its interaction with anionic heparin molecules. In this way, Col V also interacts with a number of molecules in the extracellular matrix such as proteoglycans (PG), heparin sulfate, and other triggering cellular stimulus signals (Ricard-Blum et al., 2006; Symoens et al., 2011). Col V specifically decreases endothelial cell proliferation promoting cell detachment by the disassembly of F-actin filaments, and cells started to proliferate when recultured on Col I (Mak et al., 2016). Moreover, heterozygous mutation in *Col Va2* gene could lead to connective tissue hyperelasticity and joint instability (Johnston et al., 2017). In the current study, we verified that ADSCs stimulated by Col V increased Col II synthesis, the main collagenous protein of the hyaline cartilage matrix, and decreased apoptotic chondrocytes. Based on the study from Nakajima et al. (2002a,b), who demonstrated that Col V was upregulated during adipogenesis and that it can stimulate adipocyte differentiation *in vitro*, we inferred that ADSCs stimulated by Col V enhance the expression of Col II, increasing the density of cartilage tissue. Another hypothetical translational significance of this finding is that Col II deficiency may participate in the articular deterioration progress of OA. However, how ADSCs stimulated by Col V enhancement of the expression of Col II could promote the healing process of cartilage tissue in OA needs further studies using transgenic Col V ADSCs to unveil the potential role of Col V in OA.

In the present study, we also used TGF-β1, another established growth factor for *in vitro* ADSC chondrogenic differentiation (Yarak and Okamoto, 2010) and Col II synthesis (Indrawattana et al., 2004; Shirasawa et al., 2006; Kraus, 2011; Das et al., 2015; Kwon et al., 2016), to evaluate and compare the expression of *Col2a1* and *Acan* genes in ADSC stimulated by Col V. Of note, we demonstrated similar *Col2a1* and *Acan* gene expression in ADSCs stimulated with Col V and ADSCs stimulated with TGF-β1 in relation to the control culture. The similar fold change of the expression of these genes between the two stimuli suggests once again that Col V could act as a modulator of the collagen chondrogenic. In fact, recent studies showed that ADSCs stimulated with TGF-β3 and BMP-6 had increased chondrogenic gene expressions and the model of osteoarthritic knees treated with these cells had improvement of cartilage scores (Ude et al., 2018). In addition to the application of MSCs in animal models, clinical trials and meta-analytic studies have shown cartilage improvement, reduction in pain, and improvement in joint function in patients with OA after autologous MSC injections (Centeno et al., 2008; Koh et al., 2013; Robinson et al., 2018; Huang et al., 2020). Additionally, other authors have shown that a single intra-articular injection of integrin α10β1 derived from allogeneic adipose tissue was effective in treating OA in horses, showing decreased articular cartilage fibrillation (Delco et al., 2020). Since the intra-articular cavity is closed, the loss of a few stem cells is expected. Previous studies have shown that MSCs are present up to 1 month after intra-articular injection (Satué et al., 2019), and these were being done in clinical trials (Jo et al., 2014). If stem cells are still present even after 1 month of injection, this reflects a few stem cell loss. In addition, compared with intravenous injection (Eggenhofer et al., 2012), stem cells injected in the intra-articular cavity can stay longer.

## Conclusion

In conclusion, by applying immunofluorescence, morphometry, and qRT-PCR analysis, this study demonstrated that post-ADSC stimulation by Col V treatment increased the repair process in an osteoarthritic rabbit articular cartilage model. These findings suggested that surgical-induced OA treated with ADSCs stimulated by Col V may prevent the progression of cartilage injury; nevertheless, more studies are needed in order to unveil the immunological role of ADSCs stimulated by Col V in the pathogenesis of OA.

## Data Availability Statement

The raw data supporting the conclusions of this article will be made available by the authors, without undue reservation.

## Ethics Statement

The animal study was reviewed and approved by Ethics Committee in the use of animals from Medical School of the University of São Paulo (Protocol No. 123/14).

## Author Contributions

IB contributed the methodology, data acquisition, and original draft of the manuscript. AV contributed to the methodology, draft of the manuscript and editing the manuscript, and revising the work critically for intelectual content. SC, EP, and AS contributed to the methodology. CG-S contributed to the resources. CF contributed to the data acquisition, review of the analysis, and interpretation of the data. AG contributed to the data acquisition, review of statistical analysis, and revising the work critically for intelectual content. PS and VC contributed to the review of the analysis and interpretation of data, revising the work critically for intelectual content, and final approval of the version to be published. TF, DB, and RF critically reviewed the manuscript. WT contributed to the conceptualization, draft of the manuscript, funding acquisition, data statistic analysis, and final approval of the version to be published.

## Conflict of Interest

The authors declare that the research was conducted in the absence of any commercial or financial relationships that could be construed as a potential conflict of interest.
