# Supplementary figures and images for: Post-Adipose-Derived Stem Cells (ADSC) Stimulated by Collagen Type V (Col V) Mitigate the Progression of Osteoarthritic Rabbit Articular Cartilage
Source: Front Cell Dev Biol. 2021 Mar 22;9:606890. doi: 10.3389/fcell.2021.606890 (PMC8019831; doi:10.3389/fcell.2021.606890)

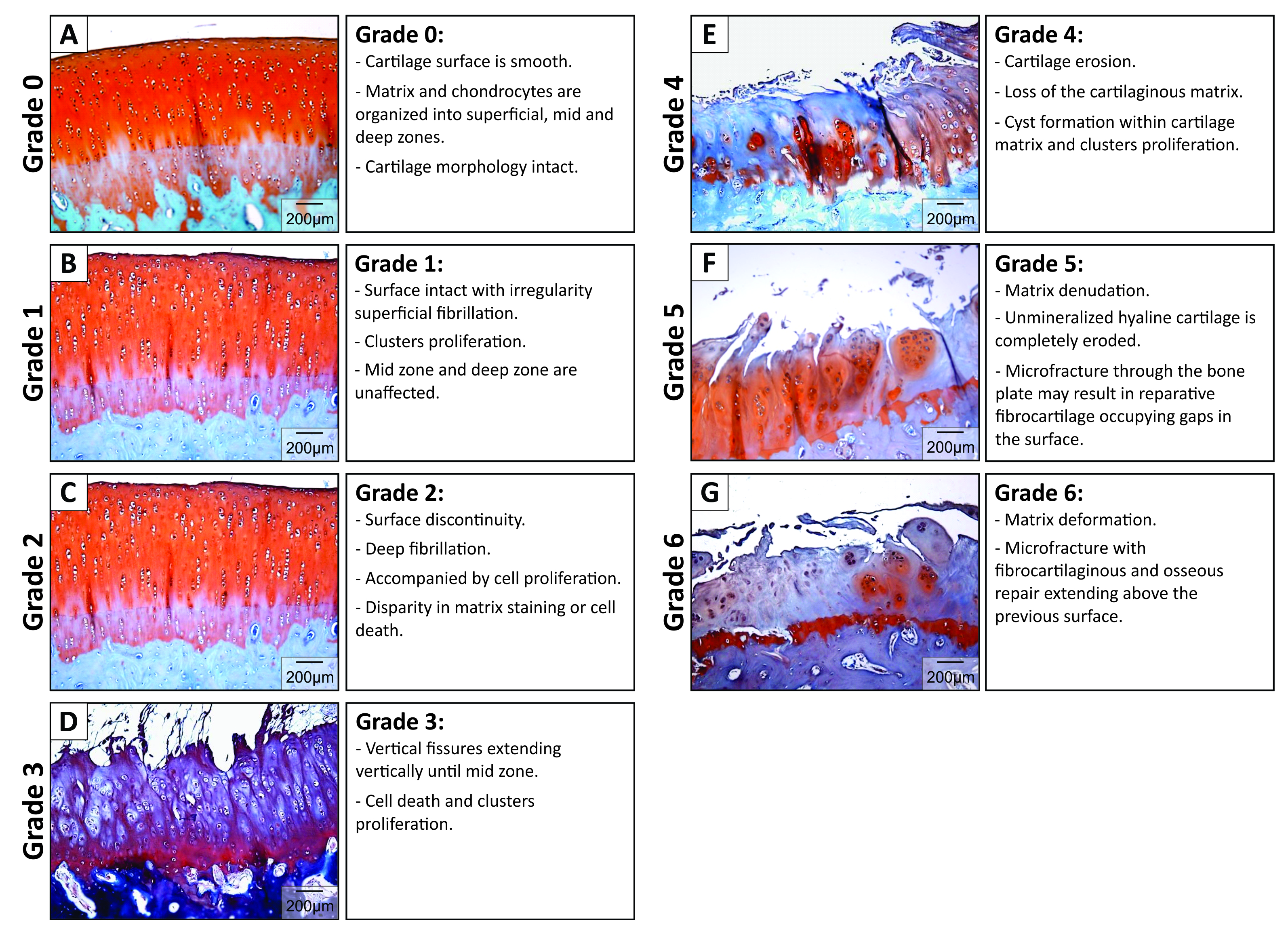

Supplement: Supplementary file 1 [file Image_1.JPEG]
